# Infant Iodine and Selenium Status in Relation to Maternal Status and Diet During Pregnancy and Lactation

**DOI:** 10.3389/fnut.2021.733602

**Published:** 2021-12-17

**Authors:** Mia Stråvik, Klara Gustin, Malin Barman, Helena Skröder, Anna Sandin, Agnes E. Wold, Ann-Sofie Sandberg, Maria Kippler, Marie Vahter

**Affiliations:** ^1^Department of Biology and Biological Engineering, Food and Nutrition Science, Chalmers University of Technology, Gothenburg, Sweden; ^2^Institute of Environmental Medicine, Karolinska Institutet, Stockholm, Sweden; ^3^Department of Clinical Science, Pediatrics, Sunderby Research Unit, Umeå University, Umeå, Sweden; ^4^Department of Infectious Diseases, Institute of Biomedicine, Sahlgrenska Academy, University of Gothenburg, Gothenburg, Sweden

**Keywords:** iodine, selenium, pregnancy, breast milk, infant, dietary intake, biomarkers, allergy

## Abstract

Iodine and selenium are essential trace elements. Recent studies indicate that pregnant and lactating women often have insufficient intake of iodine and selenium, but the impact on fetal and infant status is unclear. Here, we assessed iodine and selenium status of infants in relation to maternal intake and status of these trace elements in the birth cohort NICE, conducted in northern Sweden (*n* = 604). Iodine was measured in urine (UIC) in gestational week 29, and in breast milk and infant urine 4 months postpartum, while selenium was measured in maternal plasma and erythrocytes in gestational week 29, and in breast milk and infant erythrocytes 4 months postpartum, in both cases using ICP-MS. Maternal intake was assessed with semi-quantitative food frequency questionnaires in gestational week 34 and at 4 months postpartum. The median intake of iodine and selenium during pregnancy (98 and 40 μg/d, respectively) and lactation (108 and 39 μg/d, respectively) was below recommended intakes, reflected in insufficient status (median UIC of 113 μg/L, median plasma selenium of 65 μg/L). Also, breast milk concentrations (median iodine 77 μg/L, median selenium 9 μg/L) were unlikely to meet infant requirements. Median UIC of the infants was 114 μg/L and median erythrocyte selenium 96 μg/kg, both similar to the maternal concentrations. Infant UIC correlated strongly with breast milk levels (rho = 0.64, *p* < 0.001). Their erythrocyte selenium correlated with maternal erythrocyte selenium in pregnancy (rho = 0.38, *p* < 0.001), but not with breast milk selenium, suggesting formation of prenatal reserves. Our results indicate that the transport of iodine and selenium to the fetus and infant is prioritized. Still, it is uncertain whether most infants had sufficient intakes. Further, the results might indicate an involvement of iodine in asthma development during the first year of life, which is essential to follow up. The low maternal and infant dietary intake of both iodine and selenium, especially when the mothers did not use supplements or iodized table salt, suggest a need for a general screening of women and young children.

## Introduction

The nutritional needs of the developing fetus are met through transport of nutrients from mother to child *via* the placenta. During early infancy, these needs are covered by breast milk or by infant formula. The diet of the pregnant and lactating mother has to provide enough nutrients for fetal and infant growth and development, while still fulfilling her own needs (1). Adequate status of the nutrients iodine and selenium are essential for thyroid function (2, 3). Iodine is incorporated in the thyroid hormones triiodothyronine (T_3_) and thyroxine (T_4_,), and selenium in the iodothyronine deiodinases, which enables the conversion of T_4_ to the active thyroid hormone T_3_, as well as the deactivation of the hormones. Impaired thyroid function during pregnancy and lactation has been associated with impaired child growth and neurodevelopment, and in severe cases even increased child mortality (2–6). Selenoproteins, in which selenium is incorporated, also influence a wide range of immune responses (7), and the antioxidative abilities of some of these proteins have, for instance, been suggested to affect asthma risk (8–11). The role of iodine in allergy development is not yet known, but the sparse evidence suggests that also iodine could be involved in immune responses (12).

There is increasing evidence of insufficient iodine and selenium intake among pregnant women in Europe (13–15). Insufficient iodine intake has been suggested for Swedish pregnant women (16–18), and to a limited extent also lactating women (19), while less is known about their selenium intake. Herein we aimed to assess the iodine and selenium status of infants in relation to the dietary intake and status of their mothers during pregnancy and lactation. A secondary aim was to relate iodine and selenium intake and infant status to allergy risk at 12 months of age.

## Materials and Methods

### Study Population

This study was conducted as a part of the *Nutritional impact on Immunological maturation during Childhood in relation to the Environment* (NICE) birth cohort. Details of the cohort have previously been described (20–23). Briefly, all expecting women planning to give birth at Sunderby hospital, i.e., living in southern and eastern part of Norrbotten Region in Northern Sweden, within the recruitment period (February 2015 to March 2018), were offered to participate. Recruitment took place at a routine ultrasound around gestational week 18–19. The study was conducted in accordance with the Helsinki Declaration and approved by the Regional Ethical Review Board in Umeå, Sweden. Written consent was collected from all families, and they were informed about their right to withdraw from the study at any time point and to have their data deleted.

**Figure 1:**
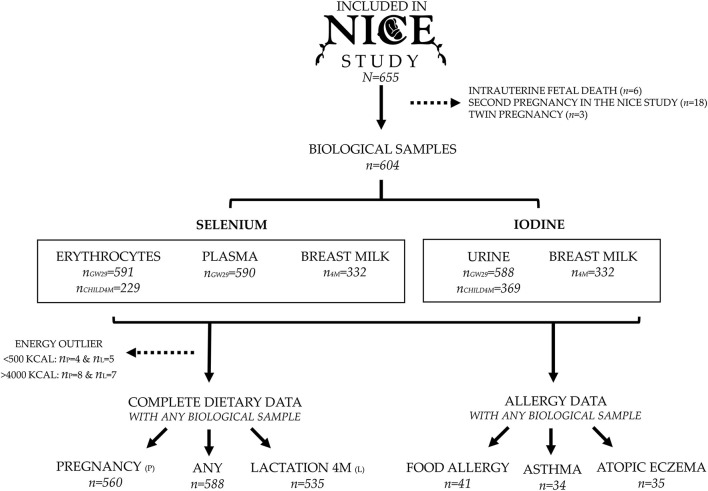
Flow chart of inclusion of pregnancies, number of available samples for measurements of iodine and selenium, and prevalence of allergic disease at 12 months of age.

A total of 655 pregnancies were included in the NICE cohort (Figure 1). Twin pregnancies (*n* = 3), pregnancies resulting in a deceased child (*n* = 6), and second pregnancies within families who participated twice (except for two cases were the first-born died) (*n* = 18), were excluded. A total of 604 mother-child pairs had an available biological sample for analysis of iodine or selenium and were therefore included in this specific study. When investigating associations between biomarkers and reported dietary intake, we excluded women without any dietary data or with an estimated energy intake outside the range of 500–4,000 kcal/d (*n* = 12), adapted from cut-offs suggested by Willet (24). This resulted in 588 mothers with valid dietary data either from pregnancy or from 4 months postpartum. Analyses of allergy risk during the first 12 months of life included 41 children with diagnosed food allergy, 34 with asthma and 35 with atopic eczema, and these cases were compared with a control group of 390 children without any allergic symptoms.

### Biomarkers and Sample Collection

Iodine concentrations were measured in spot urine samples from the mother in gestational week 29 (5−95th percentile, 27–32), and from the child at 4 months of age. Because of the short half-life of iodine (about 90% is excreted in urine within 24h) (25), urinary iodine excretion varies throughout the day (25, 26). Therefore, urinary iodine concentrations (UIC) provide information regarding iodine status solely on a group level (25). Iodine concentrations were also measured in breast milk at 4 months postpartum to assess the iodine status of the lactating woman (27) and the iodine intake of the breastfed infant. Selenium status of the mothers was assessed in gestational week 29 by concentrations in plasma as a measure of short-term intake, and in erythrocytes as a measure of more long-term intake (28). Selenium status of the infants at 4 months was assessed by concentrations in erythrocytes and the intake of the breastfed infants was assessed by selenium concentrations in breast milk.

Venous blood samples were collected from the cubital vein of the mothers at the local maternity health clinics in gestational week 29 (5−95th percentile, 27–32) (20, 21). Mothers were asked to fast for 8 h before the visit. Blood was collected in 10 mL EDTA tubes (Becton Dickinson, New Jersey, USA) and in 6 mL trace element-free Na-heparin tubes (Greiner bio-one, Kremsmünster, Austria) and stored at 4°C until transported cold to the hospital laboratory at latest the following workday. Infant venous blood samples were collected at the 4 months follow-up in 3 mL EDTA tubes (Becton Dickinson, Plymouth, UK) from the dorsal side of the infant's hand. All blood samples were centrifuged for 5 min at 2,400 rpm (Hettich Rotina 420, Hettich Lab Technology, Tuttlingen, Germany) for separation of the erythrocyte and plasma fractions.

Maternal mid-stream spot urine was collected in connection with the fasting blood sampling at the local maternity health clinics in gestational week 29 using polypropylene urine collection cups (Sarstedt Inc, Newton, NC, USA). Thus, maternal UIC was generally not influenced by peak iodine intake through meals. The samples were kept cold (4°C) until transportation to the hospital laboratory the same or the following workday, where they were transferred to 24 mL polyethylene bottles, tested free from trace elements. Infant urine was collected with urine bags, tested free from trace elements, during the 4 months follow-up visit at the hospital laboratory (20).

The mothers collected breast milk at home close to the follow-up visit at 4 months postpartum and stored it in their freezers (−20°C). They were instructed to collect (either manually or using a pump) 10 mL breast milk in each of three separate 15 mL polypropylene tubes (Sarstedt Inc, Newton, NC, USA), i.e., a total of 30 mL breast milk. The mothers were asked to collect the samples before noon, as close to an upcoming breastfeeding occasion as possible. The mothers brought the samples to the study clinic at the 4 months follow-up visit, where they were stored at −80°C. All samples were transported frozen to Karolinska Institutet, Sweden, for trace element analyses.

### Measurement of Iodine and Selenium

Concentrations of iodine [I^127^] and selenium [Se^78^] were measured by inductively coupled plasma mass spectrometry (ICP-MS; Agilent 7900, Agilent Technologies, Tokyo, Japan) equipped with an octopole reaction system (ORS). Both elements were measured in helium mode. Prior to measurement of UIC, the urine samples were diluted 1:10 with 0.1% ammonium hydroxide solution (NH_4_OH; 25% Suprapur, Merck, Darmstadt, Germany) (29). The limit of detection (LOD, three times the standard deviation of the blank concentrations) was 1.8 and 0.18 μg/L when measuring the UIC of the mothers and their infants, respectively, and no sample had a concentration below the LOD. Each run included two commercial reference materials of urine, and there was reasonable agreement between obtained and recommended values (Supplementary Tables 1, 2). The specific gravity of each urine sample (SG_sample_) was measured using a digital refractometer (EUROMEX RD712 Clinical Refractometer, Holland). To adjust for urine dilution, the infant's and the mother's UIC were adjusted to the mean urinary specific gravity (SG_mean_ 1.004 and 1.017, respectively) using the following formula: UIC_adj_ = UIC × (SG_mean_ – 1)/(SG_sample_ – 1), as previously described (30). Calculation of urinary iodine:creatinine ratio (creatinine adjustment) was not chosen, as creatinine excretion is known to be markedly affected by muscle mass, age, and meat intake (30).

Erythrocyte and plasma samples were diluted 1:25 in an alkali solution [2% (w/v) 1-butanol, 0.05% (w/v) EDTA, 0.05% (w/v) Triton X-100, 1% (w/v) NH_4_OH] and 20 μg/L internal standard (germanium, rhodium, lutetium, and iridium) and vortex mixed, then sonicated for 5 min and centrifuged at 1,000 rpm for 2 min [MSE centrifuge, Super Minor, MSE (UK) Ltd, London, England] (31). The LOD was 0.04 μg/kg for child erythrocyte selenium, and 0.6 μg/kg for maternal erythrocyte and plasma selenium. No sample had a selenium concentration below LOD. Two commercial reference materials of whole blood were included in each run of the erythrocyte samples, and two serum reference materials were included in each run of the plasma samples, and obtained values were in good agreement with the reference values (Supplementary Tables 1, 2).

Breast milk samples were diluted 1:25 in an alkali solution [2% (w/v) 1-butanol, 0.05% (w/v) EDTA, 0.05% (w/v) Triton X-100, 1% (w/v) NH_4_OH] and 100 ng/g internal standards and thereafter centrifuged at 2000 rpm for 5 min (32). The LOD was 0.3 μg/L for iodine and 0.05 μg/L for selenium, and no sample had a concentration below these limits. For quality control, one infant formula reference material and two whole blood reference materials were included in each run, and the obtained values were in good agreement with the reference values (Supplementary Tables 1, 2).

### Dietary Assessment

Maternal dietary data was collected with a semi-quantitative food frequency questionnaire (Meal-Q) sent out in gestational week 34 (mean: 34, range: 32–40), and at 4 months postpartum (mean: 16 weeks, range: 14–21 weeks postpartum). The dietary questionnaires were designed to represent intake between gestational week 30–34, and between 3 and 4 months postpartum. The dietary assessment has previously been described in detail (23). Briefly, all women received a web-based semi quantitative food frequency questionnaire, asking about intake frequency and portion sizes of food items consumed during the past month. Intake in gram per day was calculated based on reported intake frequency and estimated portion size. Based on calculated intake, food groups were created as described in detail elsewhere (22). Maternal intake of iodine and selenium was calculated in a Java-based software (MealCalc) based on reported overall diet, not including supplements (33, 34). The Meal-Q also collected information regarding regular use of multivitamin supplements with minerals. Women who reported any consumption of such supplements were asked to report the frequency of consumption with four possible replies: every day, a few times per week, a few times per month, or periodically. For this study, women were classified as supplement users if they reported a weekly or more frequent intake of supplements. Information regarding supplement brand was not collected and the absolute intake of iodine and selenium *via* supplements are not known.

Information regarding type of salt was not collected during pregnancy or lactation. Instead, when the children reached 18 months of age, the parents received a questionnaire regarding home environment and a question regarding type of salt, including iodized salt, consumed at home. This question was therefore assumed to reflect the use of salt also during pregnancy and lactation.

When evaluating the dietary intake of the infants, they were categorized as breastfed between 3 and 4 months of age if they had any available breast milk samples. Children without any available breast milk samples were defined as solely fed by infant formula. Details concerning the extent of breastfeeding (i.e., exclusively, partially, or not at all) are provided as a footnote to Supplementary Table 3. The type of infant formula used was assessed with web-based questionnaires sent out each month during the child's first year of life.

### Allergy Diagnosis

Methodology for diagnosis of allergy has previously been described in detail (22). Briefly, all families were invited to a study visit at 12 months of age to meet with the study pediatrician (author A. Sandin), who is specialized in pediatric allergology. Diagnosis of food allergy was defined as an immediate or delayed reaction after intake of a specific food with improvement once the food was excluded from the diet. The diagnosis was confirmed by a provocation causing similar symptoms. Provocation was not performed when the first reaction was acute and severe. Sensitization or specific IgE antibodies against the particular food supported diagnosis but was not mandatory. Atopic eczema was diagnosed according to William's criteria (35–37). Diagnosis of asthma was defined as any of the following: wheezing between infections, a persistent wheeze for at least 4 weeks, wheezing during an infection with concomitant allergic disease, or three episodes of wheezing during an infection, without concomitant allergic disease.

Allergic heredity and number of respiratory infections were assessed with a structured interview during the study visit at 12 months of age. The child was defined as having allergic heredity if the mother, father, or any sibling had any diagnosis of atopic eczema, food allergy, allergic rhinoconjunctivitis, and/or asthma with treatment. Information regarding respiratory infections during the first 12 months of life was collected to account for the difficulties with distinguishing allergic asthma from asthma due to respiratory infections at such an early stage in life.

### Statistical Analysis

Data analysis was performed with IBM SPSS version 26 (IBM, New York, NY, USA) and R version 3.6.2 (R Foundation for Statistical Computing, Vienna, Austria). *P*-values below 0.05 were considered significant for all tests. Distribution was investigated with the Shapiro-Wilk test of normality and cross-checked with histograms. All data were presented with median and 5-95th percentile. Associations between biomarkers (selenium in maternal plasma and erythrocytes and infant erythrocytes, iodine in urine, and selenium and iodine in breast milk) and maternal diet were investigated with Spearman correlations and visualized with heatmaps.

Associations between infants' intake of iodine and selenium (i.e., concentrations in breast milk) and concentrations in urine and erythrocytes, and associations between maternal erythrocyte concentrations and infant erythrocyte concentrations, were visualized in scatter plots with LOWESS smoothing lines.

To investigate differences in iodine and selenium status and intake between non-allergic children and allergic children (food allergy, atopic eczema, and asthma), Mann-Whitney U test was initially used, and analysis of covariance (ANCOVA) was used on significant findings in order to control for the following confounding factors which have previously been shown to be associated with childhood allergy: allergic heredity (yes/no) (38), older sibling (yes/no) (39), and season of birth (dark season, October to March; bright season, April to September) (40). As a secondary step, we explored associations of iodine and selenium intake and status with allergy outcomes using multivariable-adjusted logistic regression models. Biomarker concentrations were included as continuous variables and expressed per 10 μg. Each allergy diagnosis was included in separate models as binary outcome variables. The regression models were adjusted for the same covariates as described for the ANCOVA.

## Results

### Background Data

Background characteristics for the mothers are presented in Table 1. The median age of the mothers at the time of delivery was 30 years. In general, they had normal body weight [median body mass index (BMI) of 24 kg/m^2^] at admission to the maternity ward in the first trimester. The education level was high, most women had a university degree. Most of the women consumed an omnivore diet both during pregnancy (97%) and postpartum (96%), while 2% were vegetarians and 2% consumed seafood in an otherwise vegetarian diet during pregnancy. Multivitamin supplement with minerals (no information available on iodine and selenium content) were consumed regularly by 41% of the women during pregnancy and 18% postpartum. Iodized salt was consumed by 86% of the families, and 55% reported this to be the only salt used at home.

**Table 1:** Characteristics of included mothers categorized by low and high (median split) iodine (urinary concentrations, UIC) and selenium (plasma concentrations, P-Se) status.

**Characteristics**	***n*** **(% within group) and/or median (5−95th percentile)**						
	**All**	**UIC^1^**		***p***	**P-Se^2^**		***p***
**Mothers**	***n =* 604**	**Low *n =* 294**	**High *n =* 294**		**Low *n =* 295**	**High *n =* 295**	
**Age (years)**	30 (23–39)	30 (23–39)	30 (24–39)	0.470	30 (23–39)	30 (24–39)	0.272
*Missing*	*1*	–	*1*		–	*1*	
**Early-pregnancy BMI (kg/m^2^)**	24.3 (19.4–35.0)	24.7 (19.6–35.9)	24.3 (19.2–34.2)	0.053	24.7 (19.9–35.8)	24.2 (19.3–34.5)	0.018
Underweight (<18.5)	10 (2)	5 (2)	4 (1)	0.049^t^	5 (2)	4 (1)	0.032^t^
Normal weight (18.5–24.9)	327 (56)	148 (52)	166 (58)		144 (51)	171 (59)	
Overweight (25.0–29.9)	163 (28)	81 (28)	81 (28)		84 (29)	78 (27)	
Obesity (≥30.0)	88 (15)	53 (18)	34 (12)		52 (18)	36 (12)	
*Missing*	*16*	*7*	*9*		*10*	*6*	
**Education**							
Elementary school, 9 year	13 (2)	8 (3)	5 (2)	0.134	10 (4)	3 (1)	0.066
High school, 12 year	167 (28)	91 (31)	71 (25)	0.046^t^	85 (29)	75 (26)	0.041^t^
University or other, >12 year	416 (70)	193 (66)	212 (74)		194 (67)	215 (73)	
*Missing*	*8*	*2*	*6*		*6*	*2*	
**Smoking before pregnancy (yes)**	37 (6)	21 (7)	16 (5)	0.401^f^	21 (7)	16 (6)	0.497^f^
*Missing*	*8*	*5*	*3*		*3*	*5*	
**Vegetarian (yes)**							
Pregnancy, GW34	9 (2)	4 (1)	5 (2)	1.000^f^	8 (3)	1 (<1)	0.019^f^
*Missing*	*32*	*14*	*12*		*18*	*10*	
Postpartum, 4M	7 (1)	4 (1)	3 (1)	1.000^f^	6 (2)	1 (<1)	0.123^f^
*Missing*	*57*	*27*	*29*		*27*	*30*	
**Pescatarian (yes)**							
Pregnancy, GW34	10 (2)	1 (<1)	9 (3)	0.020^f^	2 (1)	8 (3)	0.107^f^
*Missing*	*32*	*14*	*12*		*18*	*10*	
Postpartum, 4M	14 (3)	2 (1)	11 (4)	0.012^f^	3 (1)	10 (4)	0.053^f^
*Missing*	*57*	*27*	*29*		*27*	*30*	
**Omnivores (yes)**							
Pregnancy, GW34	553 (97)	275 (98)	268 (95)	0.059^f^	267 (96)	276 (97)	0.819^f^
*Missing*	*32*	*14*	*12*		*18*	*10*	
Postpartum, 4M	526 (96)	261 (98)	251 (95)	0.072^f^	259 (97)	254 (96)	0.656^f^
*Missing*	*57*	*27*	*29*		*27*	*30*	
**Supplement use (yes)^3^**
Pregnancy, GW34	232 (41)	65 (23)	159 (56)	<0.001^f^	71 (26)	156 (55)	<0.001^f^
*Missing*	*32*	*14*	*12*		*18*	*10*	
Postpartum, 4M	100 (18)	28 (10)	70 (26)	<0.001^f^	31 (12)	68 (26)	<0.001^f^
*Missing*	*57*	*27*	*29*		*27*	*30*	
**Iodized salt use (yes)**	388 (86)	182 (84)	198 (88)	0.279^f^	192 (86)	187 (85)	0.685^f^
*Missing*	*151*	*77*	*68*		*73*	*75*	

1 *Divided into two groups with median split of maternal urinary iodine concentrations at gestational week 29. Low includes values ≤ 113.4 μg/L and high >113.4 μg/L*.

2 *Divided into two groups with median split of maternal plasma selenium concentrations at gestational week 29. Low includes values ≤ 64.73 μg/kg and high >64.73 μg/kg*.

3 *Regular use (every day or several days a week) of multivitamins with minerals. Differences in distribution between low and high groups were tested with Fisher's exact test and Pearson Chi-Square for categorical variables, and Mann-Whitney U test for continuous variables*.

t *Linear-by-linear association*.

f *Fisher's exact test*.

Background information about the infants is presented in Table 2. The median gestational age at birth was 281 days, and 47% of the infants were boys. A great majority (87%) was reported to be breastfed to any extent between 3 and 4 months of age, and 70% of these children had an available breast milk sample. Almost half of the children (49%) had an older sibling living with them, and 70% of the infants had any allergy within the family.

**Table 2:** Characteristics of included infants categorized by low and high (median split) iodine (urinary concentrations, UIC) and selenium (erythrocyte concentrations, Ery-Se) status.

**Characteristics**	***n*** **(% within group) and/or median (5–95th** **percentile)**						
	**All**	**UIC^1^**		***p***	**Ery-Se^2^**		***p***
**Infants**	***n =* 604**	**Low *n =* 185**	**High *n =* 184**		**Low *n =* 115**	**High *n =* 114**	
**Birth weight (grams)**	3565 (2720–4510)	3580 (2681–4501)	3543 (2730–4504)	0.281	3540 (2921–4568)	3575 (2524–4455)	0.887
*Missing*	*6*	–	*2*		–	*1*	
**Sex (boy)**	280 (47)	93 (50)	84 (46)	0.405^f^	58 (50)	46 (40)	0.145^f^
*Missing*	*2*	–	–		–	–	
**Gestational age at birth (days)**	281 (260–295)	281 (261–295)	281 (254–295)	0.985	283 (262–297)	281 (260–294)	0.188
*Missing*	*5*	–	*2*		–	–	
**Season of birth**							
October to March	277 (46)	85 (46)	89 (49)	0.602^f^	54 (47)	37 (33)	0.031^f^
April to September	321 (54)	100 (54)	93 (51)		61 (53)	76 (67)	
*Missing*	*6*	–	*2*		–	*1*	
**≥1 older sibling at home (yes)**	298 (49)	92 (50)	90 (49)	0.917^f^	54 (47)	54 (47)	1.000^f^
**Allergic heredity (yes)**	358 (70)	126 (74)	119 (68)	0.239^f^	74 (69)	67 (62)	0.316^f^
*Missing*	*90*	*14*	*8*		*8*	*6*	
**Allergy, 12 months (yes)**							
Food allergy	41 (7)	11 (6)	14 (8)	0.677^f^	11 (10)	7 (6)	0.326^f^
Atopic eczema	35 (6)	11 (6)	10 (5)	1.000^f^	11 (10)	7 (6)	0.326^f^
Asthma	34 (6)	11 (6)	12 (7)	1.000^f^	3 (3)	5 (4)	0.724^f^
*Missing^3^*	*105*	*18*	*12*		*12*	*8*	
**Breastfeeding^4^**							
None	71 (13)	13 (7)	34 (20)	<0.001^t^	16 (14)	17 (16)	0.619^t^
Partially	117 (22)	33 (19)	38 (23)		27 (24)	18 (17)	
Exclusively	342 (65)	128 (74)	96 (57)		68 (61)	73 (68)	
*Missing*	*74*	*11*	*16*		*4*	*6*	

1 *Divided into two groups with median split of urinary iodine concentrations at 4 months of age. Low includes values ≤ 113.8 μg/L and high >113.8 μg/L*.

2 *Divided into two groups with median split of erythrocyte selenium concentrations at 4 months of age. Low includes values ≤ 96.51 μg/kg and high >96.51 μg/kg*.

3 *Uncertain diagnoses were coded as missing, meaning the number of missing observations is not due to lack of data*.

4 *Extent of breastfeeding at the time of sampling (i.e., between three and 4 months of age)*.

*Differences in distribution between low and high groups were tested with Fisher's exact test and Pearson Chi-Square for categorical variables, and Mann-Whitney U test for continuous variables*.

t *Linear-by-Linear association*.

f *Fisher's exact test*.

### Maternal Dietary Intake of Iodine and Selenium During Pregnancy and Lactation

**Figure 2:**
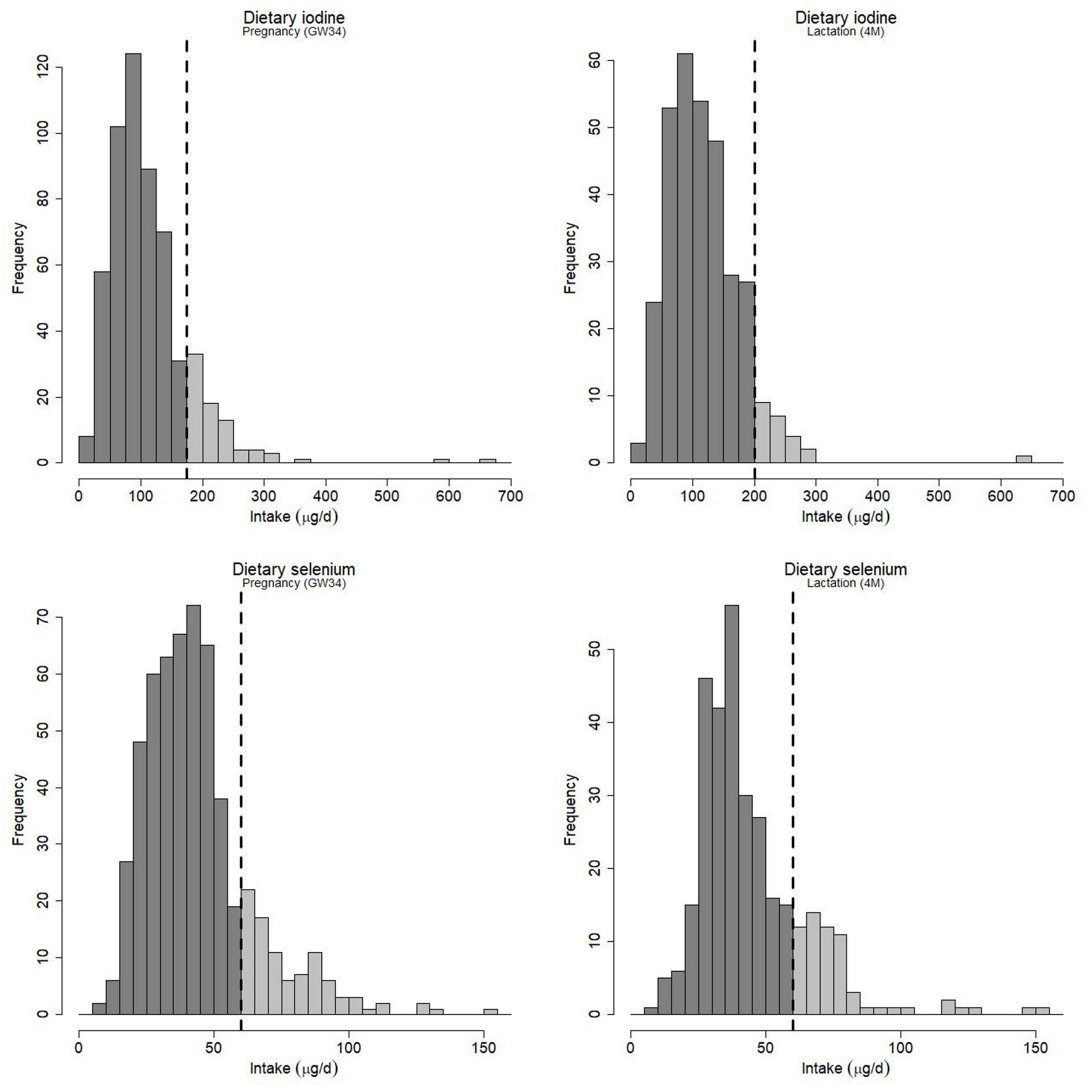
Estimated maternal intake of iodine and selenium from dietary sources during gestational week 34 (GW34) and during lactation at 4 months postpartum (4M). Intake from supplements is not included. The vertical dashed line represents the recommended daily intake for pregnant and lactating women as given by the Nordic Council of Ministers (41).

Figure 2 shows the distributions of calculated dietary intake of iodine (iodine in salt and supplements not included) during the third trimester [median (5-95th percentiles): 98 (38–225) μg/d] and during lactation [108 (39–213) μg/d]. The intake at 4 months postpartum among women without any breast milk sample (i.e., classified as not breastfeeding, *n* = 214) was similar, 101 (35–214) μg/d. Only 14% of the pregnant women had an intake at or above the recommended daily intake (RDI) of iodine [175 μg/d, according to the Nordic Council of Ministers (41)] and only 7% of the breastfeeding women had a sufficient intake during lactation (RDI 200 μg/d).

Similarly, the intake of selenium was low, the median (5-95th percentiles) intake was 40 (19–85) μg/d in the pregnant women, 39 (22-78) μg/d in the breastfeeding women, and 36 (17–90) μg/d among women who did not breastfeed their infants at 4 months postpartum. As can be seen in Figure 2, about 17% of the women during pregnancy, and 19% of the women during lactation, had a selenium intake at or above the recommended level, which is 60 μg/d during both pregnancy and lactation (41). Hence, only 5% had an adequate intake of both iodine and selenium during pregnancy, while corresponding fraction during lactation was 3%.

### Maternal and Infant Iodine and Selenium Status

Table 3 shows the concentrations of maternal and infant UIC and selenium in blood fractions. The women's UIC was low during pregnancy with a median of 113 μg/L (adjusted to mean specific gravity), which is below the 150 μg/L as is considered to reflect adequate intake on a population level by the WHO (42). Women taking supplements had considerably higher UIC (152 μg/L), which was also reflected in their breast milk (100 μg/L compared to 74 μg/L in women not taking supplements). As shown in Table 1, women with above median UIC were more likely to have a university education, less likely to be obese, and were more frequently pescatarians (consuming seafood in an otherwise vegetarian diet) both during pregnancy and postpartum than women with UIC below median.

**Table 3:** Biomarker iodine and selenium concentrations in gestational week 29 (GW29), gestational week 34 (GW34), and at 4 months postpartum (4M) for all included mothers and infants and grouped by use of multivitamin supplement with minerals.

**Biomarker**	***n***	**All Median (5–95th)**	***n***	**Supplement Median (5−95th)**	***n***	**No supplement Median (5−95th)**	**p**
**IODINE**							
Maternal urine, μg/L (GW29)^1^	588	113 (53.5–313)	224	152 (61.6–346)	338	98.6 (49.3–204)	<0.001
Breast milk, μg/kg (4M)	332	76.6 (32.6–204)	63	100 (39.1–213)	263	74.2 (31.9–200)	0.007
Infant urine, μg/L (4M)^2^	369	114 (47.9–243)	60	137 (48.0–335)	293	110 (47.8–220)	0.002
Breastfed^3^	222	110 (46.1–248)	38	144 (42.0–352)	179	106 (45.9–219)	<0.001
Not breastfed^3^	147	117 (58.2–237)	22	114 (68.5–421)	114	115 (57.7–228)	0.727
**SELENIUM**							
Maternal plasma, μg/kg (GW29)	590	64.7 (44.9–91.0)	227	69.6 (49.5–96.0)	335	61.9 (42.6–89.4)	<0.001
Maternal erythrocytes, μg/kg (GW29)	591	106 (78.3–146)	227	109 (80.8–148)	335	104 (76.3–145)	0.002
Breast milk, μg/kg (4M)	332	9.00 (5.73–13.5)	63	9.36 (6.07–13.7)	263	8.92 (5.70–13.3)	0.017
Infant erythrocytes, μg/kg (4M)	229	96.5 (74.0–134)	43	94.0 (71.2–153)	178	97.3 (73.4–132)	0.915
Breastfed^3^	138	95.7 (73.2–134)	33	94.0 (73.3–147)	104	96.4 (71.1–134)	0.960
Not breastfed^3^	91	97.0 (77.0–134)	10	94.7 (84.3–134)^4^	74	97.8 (77.4–129)	0.740

*Differences between supplement group and non-supplement group were tested with Mann-Whitney U test. Supplement use was defined as regular consumption (every day or several days a week) of multivitamins with minerals. One individual can be present in supplement group during pregnancy, but in non-supplement group during lactation*.

1 *Adjusted to the mothers mean urinary specific gravity (SG_mean_ = 1.017)*.

2 *Adjusted to the infants mean urinary specific gravity (SG_mean_ = 1.004)*.

3 *Individuals with available breast milk samples at 4 months were defined as being breastfed*.

4 *25−75th Percentile*.

The median UIC of the 4 months old infants (114 μg/L, Table 3) was similar to the UIC of the mothers. Breastfed infants tended to have lower median UIC than those solely given formula (110 vs. 117 μg/L, *p* = 0.081). Maternal supplementation appeared to improve infant UIC among the breastfed infants (144 vs. 106 μg/L, *p* < 0.001), although few of the lactating women (18%) reported regular use of supplements between 3 and 4 months postpartum.

As shown in Table 3, the median concentration of selenium in maternal plasma and erythrocytes was 65 and 106 μg/kg, respectively. The selenium concentration in breast milk was much lower with a median of 9 μg/kg. Maternal use of supplements at the time of blood sampling was reflected in 12% higher plasma selenium concentrations, compared to those who did not use supplements, and to some extent also with higher concentrations in erythrocyte and breast milk (both 5% higher). Women with above median plasma selenium concentrations were more likely to have a university education, less likely to be obese, and they more frequently reported a regular use of supplements, than women with lower concentrations (Table 1).

The concentration of erythrocyte selenium in the 4 months old infants was almost as high (median 97 μg/kg) as in the mothers (Table 3). Breastfed infants had similar concentrations as those receiving infant formula (96 vs. 97 μg/kg, *p* = 0.88), and infants of mothers taking supplements had similar selenium concentrations as those not taking supplements (94 and 97 μg/kg, respectively). As indicated in Table 2, the infants born in April to September had higher erythrocyte selenium concentrations than those born in October to March [median (5−95th percentiles): 98 (76–135) vs. 93 (67–133) μg/kg, *p* = 0.007].

### Correlations Between Different Markers of Iodine and Selenium

**Figure 3:**
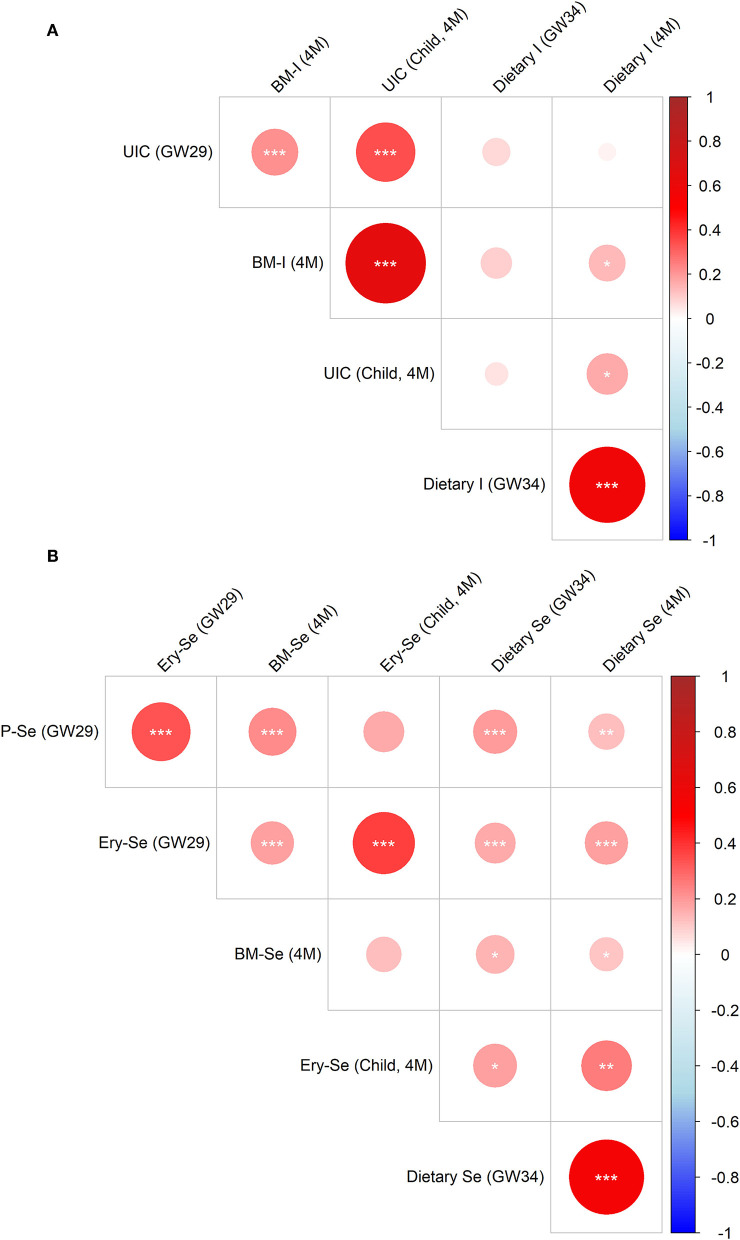
Correlation matrix of iodine **(A)** and selenium **(B)** concentrations in erythrocytes (Ery), plasma (P), urine (U), and breast milk (BM) and estimated intake from diet in gestational week 34 (GW34) and 4 months postpartum (4M) among all included mothers and their breastfed infants. Significant correlations are denoted with asterisks as follows: *p* < 0.001 =^***^, *p* < 0.01 =^**^ and *p* < 0.05 =^*^.

Correlations between different biomarkers (i.e., plasma, erythrocytes, urine, and breast milk) of iodine and selenium and estimated intake of iodine and selenium from the total diet are presented in Figure 3. The estimated maternal intake levels of iodine during pregnancy and lactation were linked (rho = 0.57, *p* < 0.001; Figure 3A). Maternal UIC during pregnancy correlated weakly with breast milk concentrations (rho = 0.22, *p* < 0.001), despite the seven months in between the sampling occasions. The breastfed infants' UIC, however, correlated strongly with the breast milk iodine concentrations (rho = 0.64, *p* < 0.001; Supplementary Figure 1).

Regarding selenium, maternal intake during pregnancy and lactation correlated strongly (rho = 0.55, *p* < 0.001; Figure 3B). Maternal plasma and erythrocyte selenium concentrations correlated moderately (rho = 0.34, *p* < 0.001), while plasma (gestational week 29) and breast milk selenium correlated weakly (rho = 0.22, *p* < 0.001). The breastfed infants' selenium concentrations at 4 months of age (measured in erythrocytes) did, however, not correlate with breast milk selenium concentrations (rho = 0.12, *p* = 0.15), but moderately with maternal erythrocyte selenium concentrations during pregnancy (rho = 0.38, *p* < 0.001).

### Relation Between Maternal Food Intake and Iodine and Selenium Levels in Mother and Infant

**Figure 4:**
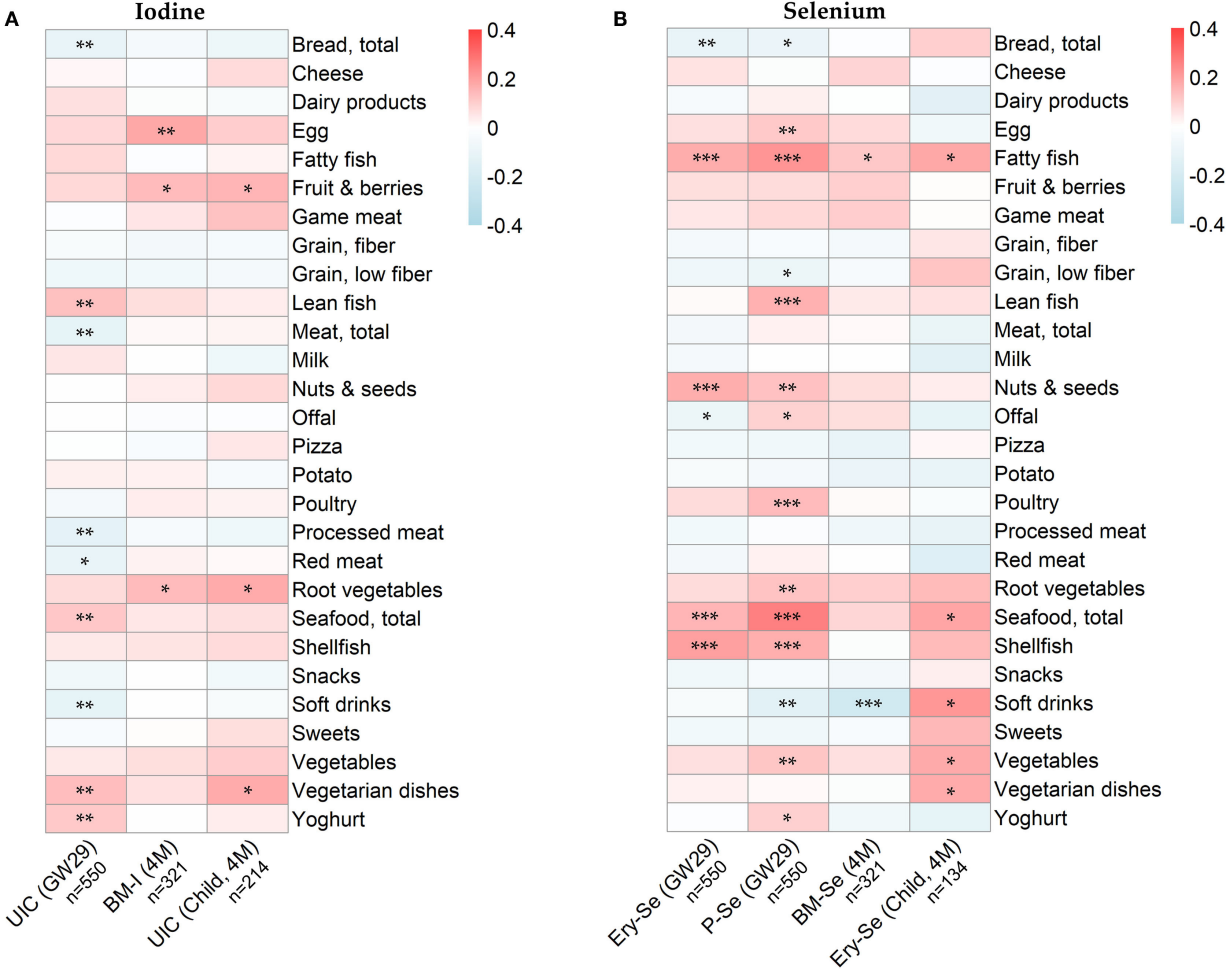
Heatmaps displaying Spearman correlations between **(A)** iodine concentrations in urine (UIC) and breast milk (BM-I), and maternal food intake **(B)** selenium concentrations in erythrocytes (Ery-Se), plasma (P-Se) and breast milk (BM-Se), and maternal food intake. Food intake was assessed to reflect maternal intake in gestational week 30–34 and at 3–4 months postpartum. Associations between infants' iodine and selenium concentrations and maternal food intake was investigated solely for breastfed infants (i.e., with available breast milk samples). Significant correlations are denoted with asterisks as follows: *p* < 0.001 =^***^, *p* < 0.01 =^**^ and *p* < 0.05 =^*^.

To identify specific food sources contributing to iodine and selenium status, both of the mother and of the child, we analyzed correlations between maternal food intake (assessed in gestational week 34 and at 4 months postpartum) and the different biomarkers. As shown in Figure 4A, food sources associated with higher maternal UIC included lean fish, total seafood, vegetarian dishes, and yogurt, while food sources associated with lower UIC were total bread, total meat, processed meat, red meat, and soft drinks. During lactation, a higher intake of egg, fruits and berries and root vegetables were associated with higher breast milk iodine.

A higher intake of fatty fish, nuts and seeds, shellfish and total seafood was associated with higher maternal erythrocyte selenium concentrations (Figure 4B). Even more associations could be seen with maternal plasma selenium concentration, for which also lean fish, offal, poultry, root vegetables, vegetables and yogurt were associated with higher concentrations. The diet of the lactating mothers was, however, neither well reflected in breast milk selenium concentrations nor in infant erythrocyte selenium, except for fatty fish.

Since concentrations of iodine and selenium in blood and breast milk were higher in supplement users than in women not using supplements, sub-analyses were conducted solely with the women not reporting any regular supplement use. Associations between dietary intake and the different biomarkers among non-supplement users did not differ markedly from the main results which included all women (Supplementary Figure 2).

The use of iodized salt influenced both maternal and infant UIC (Table 4). Women who reported iodized table salt as the single source of added salt (*n* = 247) had significantly higher UIC during pregnancy as compared to women who never consumed iodized table salt (*n* = 65) (117 vs. 106 μg/L, *p* = 0.027). The difference in breast milk concentrations was also apparent (78 vs. 53 μg/L), although not statistically significant, probably because we had milk samples from only 37 women who never consumed iodized table salt. Also, the UIC of the breastfed children in families using iodized table salt were significantly higher as compared to those who never used iodized table salt (115 vs. 85 μg/L, *p* = 0.026).

**Table 4:** Biomarker iodine concentrations in gestational week 29 (GW29) and at 4 months postpartum (4M) grouped by the use of iodized table salt.

**Biomarker**	***n***	**Non-iodized table salt *and/or* non-table salt^1^ (*n =* 65)**	***n***	**Mix of iodized table salt *and* other (*n =* 141)**	***n***	**Solely iodized table salt (*n =* 247)**	***p***
		**Median (25−75th)**		**Median (25−75th)**		**Median (25–75th)**	
**IODINE**							
Maternal urine, μg/L (GW29)^2^	63	106 (74–134)^a^	138	113 (81–175)	242	117 (83–176)^a^	0.095
Breast milk, μg/kg (4M)	37	53 (40–117)	90	73 (50–115)	154	78 (54–119)	0.245
Infant urine, μg/L (4M)^3^	44	102 (58–146)	85	115 (92–145)	170	115 (81–155)	0.221
Breastfed	28	85 (51–144)^a^	53	114 (85–142)	108	115 (80–171)^a^	0.074
Not breastfed^4^	16	117 (85–161)	32	117 (95–153)	62	114 (87–137)	0.739

*Differences between salt groups were tested with Kruskal-Wallis test and presented with p-values. Test between two groups per time was made with Mann-Whitney U test and significant differences between groups are presented with matching letters in superscript*.

1 *Non-Table Salt includes use of Flake Salt/Coarse sea Salt, Rock Salt (e.g., Himalayan Salt), Mineral Salt (low in Sodium e.g., Seltin) or Other Salt*.

2 *Adjusted to the mothers mean urinary specific gravity (SG_mean_ = 1.017)*.

3 *Adjusted to the infants mean urinary specific gravity (SG_mean_ = 1.004)*.

4 *Estimated based on available breast milk sample at 4 months or not, regardless of reported extent*.

### Allergy Diagnosis at 12 Months of age in Relation to Biomarkers

The prevalence of food allergy, atopic eczema and asthma was similar between the low and high groups of infant UIC and erythrocyte selenium concentrations at 4 months of age (Table 2). However, mothers to children with asthma had lower reported iodine intakes at 4 months postpartum than mothers to children without any allergy (81 vs. 112 μg/d, *p* = 0.012, Supplementary Table 4). This difference remained significant also after adjusting for confounding factors (*p* = 0.013).

To further investigate the potential role of selenium and iodine status and dietary intake on allergy at 12 months of age, multivariable-adjusted logistic regression analyses were conducted. Higher maternal dietary intake of iodine at 4 months postpartum was significantly associated with lower odds of infant asthma during the first year of life, both in the unadjusted (OR = 0.91, 95% CI: 0.83-0.98) and in the adjusted model (OR = 0.90, 95% CI: 0.82–0.97) (Table 5). Also, maternal iodine intake and UIC during pregnancy and breast milk iodine were inversely associated with odds of infant asthma, even though these associations were not statistically significant. In total 88% of the children with asthma diagnosis had three or more respiratory infections during the first year of life, and all had at least one infection, highlighting the difficulties with diagnosing allergic asthma at such an early stage in life (i.e., difficulties distinguishing allergic asthma from asthma due to respiratory infections).

**Table 5:** Logistic regression analyses of iodine status with infant allergy diagnosis at 12 months of age.

**Biomarker or intake *per 10 μg***	***n***	**Food allergy OR (95% CI)**	***p***	***n***	**Atopic eczema OR (95% CI)**	***p***	***n***	**Asthma OR (95% CI)**	***p***
**UNADJUSTED**									
Dietary intake, μg/d (GW34)	38^1^/370^2^	0.94 (0.88–1.01)	0.11	32/370	1.00 (0.93–1.06)	0.89	32/370	0.96 (0.88–1.02)	0.22
Maternal urine, μg/L (GW29)	38/380	1.03 (0.99–1.06)	0.10	33/380	1.03 (0.99–1.07)	0.07	34/380	0.95 (0.89–1.01)	0.12
Dietary intake, μg/d (4M)	37/373	0.94 (0.87–1.00)	0.07	31/373	0.99 (0.92–1.05)	0.68	32/373	0.91 (0.83–0.98)	0.02
Breast milk, μg/kg (4M)	23/256	1.01 (1.00–1.04)	0.19	20/256	0.95 (0.85–1.03)	0.32	16/256	0.89 (0.76–1.00)	0.10
Infant urine, μg/L (4M)	25/266	0.99 (0.93–1.04)	0.85	21/266	1.00 (0.93–1.05)	0.91	23/266	0.99 (0.93–1.04)	0.82
**ADJUSTED^3^**									
Dietary intake, μg/d (GW34)	38/368	0.94 (0.88–1.01)	0.10	32/368	1.00 (0.93–1.06)	0.89	31/368	0.95 (0.87–1.02)	0.15
Maternal urine, μg/L (GW29)	38/378	1.03 (0.99–1.06)	0.11	33/378	1.03 (0.99–1.07)	0.09	33/378	0.96 (0.90–1.01)	0.17
Dietary intake, μg/d (4M)	37/372	0.94 (0.87–1.00)	0.07	31/372	0.99 (0.92–1.05)	0.71	31/372	0.90 (0.82–0.97)	0.01
Breast milk, μg/kg (4M)	23/256	1.01 (1.00–1.06)	0.17	20/256	0.96 (0.86–1.04)	0.39	16/256	0.89 (0.76–1.00)	0.11
Infant urine, μg/L (4M)	25/265	1.00 (0.93–1.05)	0.90	21/265	1.00 (0.93–1.06)	0.98	22/265	0.98 (0.91–1.04)	0.63

*Concentrations were included in the models as continuous explanatory variables and the odds ratios are calculated for an increase of 10 μg*.

1 *Number of children with allergy diagnosis*.

2 *Number of children without allergy diagnosis*.

3 *All models were adjusted for the covariates: allergic heredity (yes/no), older sibling (yes/no) and season of birth (dark/bright)*.

Neither selenium intake nor biomarker concentrations of selenium of the mothers or the infants were significantly associated with infant allergy risk during the first year of life (Table 6).

**Table 6:** Logistic regression analyses of selenium status with infant allergy diagnosis at 12 months of age.

**Biomarker or intake *per 10 μg***	***n***	**Food allergy OR (95% CI)**	***p***	***n***	**Atopic eczema OR (95% CI)**	***p***	***n***	**Asthma OR (95% CI)**	***p***
**UNADJUSTED**									
Dietary intake, μg/d (GW34)	38^1^/370^2^	0.90 (0.74–1.08)	0.30	32/370	0.89 (0.71–1.08)	0.28	32/370	1.04 (0.87–1.21)	0.67
Maternal plasma, μg/kg (GW29)	39/380	1.16 (0.92–1.46)	0.19	33/380	1.08 (0.84–1.38)	0.53	34/380	1.10 (0.85–1.40)	0.47
Maternal erythrocytes, μg/kg (GW29)	40/379	0.98 (0.84–1.13)	0.74	34/379	0.98 (0.84–1.15)	0.85	34/379	1.00 (0.85–1.17)	1.00
Dietary intake, μg/d (4M)	37/373	0.95 (0.78–1–11)	0.54	31/373	1.03 (0.86–1.21)	0.73	32/373	0.90 (0.72–1.08)	0.30
Breast milk, μg/kg (4M)	23/256	1.00 (0.83–1.15)	0.99	20/256	0.95 (0.76–1.12)	0.61	16/256	1.07 (0.90–1.22)	0.35
Infant erythrocytes, μg/kg (4M)	18/166	0.83 (0.62–1.10)	0.22	18/166	0.86 (0.64–1.13)	0.28	8/166	1.22 (0.83–1.76)	0.30
**ADJUSTED^3^**									
Dietary intake, μg/d (GW34)	38/368	0.90 (0.73–1.07)	0.27	32/368	0.88 (0.70–1.08)	0.26	31/368	1.02 (0.84–1.20)	0.84
Maternal plasma, μg/kg (GW29)	39/378	1.19 (0.94–1.49)	0.14	33/378	1.12 (0.87–1.43)	0.37	33/378	1.11 (0.86–1.43)	0.40
Maternal erythrocytes, μg/kg (GW29)	40/377	0.99 (0.84–1.15)	0.88	34/377	1.01 (0.85–1.19)	0.88	33/377	1.00 (0.83–1.18)	0.96
Dietary intake, μg/d (4M)	37/372	0.96 (0.79–1.12)	0.62	31/372	1.04 (0.86–1.22)	0.67	31/372	0.87 (0.69–1.06)	0.21
Breast milk, μg/kg (4M)	23/256	1.01 (0.83–1.17)	0.95	20/256	0.95 (0.76–1.15)	0.64	16/256	1.07 (0.88–1.23)	0.41
Infant erythrocytes, μg/kg (4M)	18/165	0.89 (0.65–1.17)	0.42	18/165	1.03 (0.76–1.38)	0.86	8/165	1.22 (0.84–1.78)	0.29

*Concentrations were included in the models as continuous explanatory variables and the odds ratios are calculated for an increase of 10 μg, with the exception for concentrations in breast milk which were investigated per μg*.

1 *Number of children with allergy diagnosis*.

2 *Number of children without allergy diagnosis*.

3 *All models were adjusted for the covariates: allergic heredity (yes/no), older sibling (yes/no) and season of birth (dark/bright)*.

## Discussion

Here we describe maternal intake of selenium and iodine during pregnancy and lactation, relate it to the nutritional status of the mothers, assessed by concentrations in blood and urine, and then follow the transport of the nutrients *via* breast milk to the infants. We found that most of the women in the NICE cohort had insufficient dietary intake of iodine and selenium, both during pregnancy and lactation, which was reflected as insufficient status, as estimated based on maternal UIC and plasma selenium. Also, the concentrations in breast milk were low and breastfed infants appeared to have inadequate intake of iodine and selenium at 4 months of age. Importantly, the ways in which the infant's status was influenced by the mother appeared to differ for iodine and selenium, with the latter being transferred mainly prenatally.

Most of the mothers in the NICE cohort (about 90%) had dietary intake levels of iodine below the RDI of the Nordic Council of Ministers (41) during both pregnancy (median 98 μg/d vs. the RDI of 175 μg/d) and lactation (108 μg/d vs. the RDI of 200 μg/d). A low dietary intake of iodine in Sweden was recognized already in the 1930s because of a high prevalence of goiter, and iodized table salt was therefore introduced (16). However, also the measured UIC of the mothers during pregnancy in the present study indicated inadequate iodine status [UIC median 113 μg/L compared to recommended 150–249 μg/L (42)], why there is good reason to believe that many of the women had an insufficient total intake of iodine. In support, an even lower median UIC (101 μg/L, *n* = 737) was noted in a recent nation-wide study of pregnant women (17). To note, only 55% of the women used iodized salt as the only table salt at home and these women had higher UIC (117 μg/L) than those using unfortified salt (106 μg/L). Still, the median UIC of the mothers using iodized table salt did not reach the suggested lower median level for adequate intake. A reduction in the total salt intake, along the lines of the ongoing global discussion (43), could therefore raise concerns regarding the iodine intake of pregnant and lactating women in areas depending on iodized table salt. Given that the women in our study were older, of higher education level, and more frequently used dietary supplements, as compared to non-participating women giving birth at the same hospital during the recruitment period (44), it is likely that the iodine and selenium status is even more compromised than indicated in this study.

We also measured the iodine concentrations in breast milk and urine of the infants at 4 months of age. The mammary gland concentrates iodine through the sodium-iodide symporter (NIS), which is up-regulated during lactation (45, 46). The iodine concentration in breast milk is 20–50 times that in plasma, which implies that the uptake of iodine by the mammary gland is highly prioritized (47). The median concentration of iodine in breast milk of the NICE mothers was 77 μg/kg, which is in the lower range in an international perspective (47), and unlikely to provide the recommended 90 μg/d for all infants (45). We found a strong correlation between iodine concentrations in breast milk and infant urine, and the UIC of the breastfed infants (median 110 μg/L) indicated sufficient iodine intake on a group level, according to the currently suggested median UIC of ≥100 μg/L by WHO (42). However, using the proposed relationship between iodine intake and UIC in infants (assuming an excretion of 90% into urine and a daily urine volume of 300–500 ml) (45, 48) the recommended minimum intake of 90 μg/d would correspond to 180–225 μg/L in infant urine (48). Thus, it may be questioned if the infants indeed had a sufficient iodine intake. This uncertainty, as well as the finding that breastfed infants of mothers who did not use iodized table salt, although rather few, did not even reach a median UIC of 100 μg/L, emphasize the need to follow up the infants' iodine status as the iodine concentrations in breast milk decrease gradually during lactation (46).

Importantly, use of multivitamin supplement with minerals appeared to be more efficient than iodized table salt in providing iodine. It was associated with 35–50% higher iodine concentrations in both maternal urine, breast milk, and urine of the breastfed infants, in line with previous studies (46, 49). In fact, the women regularly taking supplements was the only group with sufficient iodine status, as indicated by their median UIC of 152 μg/L. Notably, children who were formula fed had slightly higher UIC than the children who were breastfed at least to some extent, and the infants who were exclusively breastfed had the lowest UIC (Supplementary Table 3). The most common brand of infant formula in our study contains 13 μg iodine per 100 ml according to the manufacturer. An ingested volume of 700–1,000 ml/d (producer's recommendation) equals 91–130 μg iodine per day, as compared to 54–77 μg in exclusively breastfed infants, assuming a similar milk intake. In line with these findings, a recent systematic review showed generally higher UIC in formula fed than in breastfed infants in both iodine-deficient countries (overall 70 and 38 μg/d, respectively) and iodine-sufficient countries (310 and 164 μg/d, respectively) (50). However, when estimating the infants' iodine intake based on their median UIC (45, 48), the formula fed infants still got less (about 40–70 μg/d) than the recommended intake of 90 μg/d.

The primary dietary sources contributing to the iodine status during pregnancy, based on correlation with UIC, appeared to be seafood and yogurt. Seafood is a known source of iodine [about 180 μg/100 g cod and 20 μg/100 g cultivated salmon (51)] and the women in our study consumed about 26 g seafood per day. Despite the low density of iodine in Swedish yogurt [about 8 μg/100 g natural yogurt with 3% fat (51)], it contributed significantly to iodine status, which can be explained by the relatively high yogurt consumption in Sweden (median 100 g/d in our study). Some countries have chosen to fortify cow's fodder with iodine as a strategy for combating iodine deficiency, whereas in others it is mandatory to use iodized salt in, for instance, commercial bread (25, 52). The use of iodized salt in the Swedish food industry is voluntarily and rarely applied. Also, much of the salt used in international food production is not iodized (25). Hence, both bread and processed meat were negatively associated with maternal UIC.

The strongest association between maternal diet and iodine concentrations in breast milk was seen for intake of eggs, which like breast milk have the unique capacity to concentrate nutrients required for early growth and development (53, 54). Hence, egg is dense in iodine (42 μg/100 g) (51). Significant associations were also found with fruits and berries, and root vegetables, as reflected by iodine in both breast milk and infant urine, but neither fruits and berries nor root vegetables are rich in iodine. However, supplement users were found to consume more fruit and berries than women not taking multivitamin supplements regularly (258 g/d vs. 161 g/d, *p* = 0.007). In addition, intake of fruit and berries correlated with the intake of iodine-rich food sources such as total seafood (rho = 0.266, *p* < 0.001).

The frames for adequacy of selenium status are not as fixed as for iodine and estimated adequate levels are primarily based on the functionality of selenoproteins (28). For instance, optimum activity of glutathione peroxidase (GPx) has in several studies been detected at plasma selenium concentrations around 90 μg/L (28). In our study, the median plasma selenium concentration during pregnancy was close to 65 μg/L, slightly lower than what was found in a previous study in the middle part of Sweden (72 μg/L) (54). The concentrations are in line with the finding that most of the women in fact had lower intake (median 40 μg/d) than recommended (60 μg/d) (41). Median selenium levels in breast milk at 4 months postpartum was 9 μg/L, which is low when put in a global context (55–57). It is also lower than the median of 12–13 μg/L previously reported in Sweden (58, 59), indicating regional differences in selenium intake or a decreasing intake over time. The measured breast milk concentrations of selenium provided the infants with about 7–9 μg/d, i.e., less than the suggested need of 10–15 μg/d (56, 60, 61). Interestingly, the selenium concentrations in infant erythrocytes did not correlate with the selenium content of the breast milk, but rather to the mother's erythrocyte selenium, indicating that a major part of the infant selenium was provided during fetal life as previously proposed (57, 62, 63). Accordingly, the season of birth impacted selenium (but not iodine) status of the infants, with higher selenium concentrations in those born in April to September and lower in those born in October to March. This difference indicates a seasonal variation in maternal selenium intake during pregnancy, which has previously been described (64).

Whether the infants' selenium status really was adequate is not clear. There are no functional criteria that reflect required intake of selenium. Recent studies in Norway showed that lower dietary intake of selenium during pregnancy (median 53 μg/d, i.e., slightly higher than in the present study) was associated with increased risk of preterm delivery and impaired fetal growth (15, 65). Further, a study of Bangladeshi women with similar selenium status as in the present study found a positive association between selenium concentrations in maternal erythrocytes and child cognitive function at 1.5–10 years of age (6, 66). Even if the infant selenium status would be adequate after birth, the reserves obtained prenatally are limited and the concentration in breast milk declines over time (57), suggesting that selenium levels should be followed-up at an older age.

Maternal intake of fish and seafood was associated with increased blood selenium concentrations, especially in the mothers. Seafood is a known dietary source of selenium, containing primarily selenomethionine which is highly bioavailable (67). Associations were also identified with consumption of egg and poultry. Eggs are dense in selenium (23 μg/100 g) (51), and some poultry feed is enriched with selenium, which have been found to effectively raise the concentrations also in the hen (68). The intake of selenium from the most consumed infant formula in our study can be estimated to 18–26 μg/d. Interestingly, even though this is substantially higher than what the breastfed infants consumed (6–9 μg/d), the concentration of erythrocyte selenium did not differ between breastfed and formula fed children. The result may reflect a difference in bioavailability of selenium depending on the chemical form used in the infant formula (69).

The children were assessed for allergy at 12 months of age. At this early age, food allergy and eczema are the major allergic manifestations while asthma and allergic rhinitis usually present later on. While there was no relation between iodine or selenium status and infant allergy diagnosis, we noted that a higher maternal intake of iodine from dietary sources during lactation was associated with lower odds of the child developing asthma during the first year of life. Iodine is crucial for thyroid function (70), and hypothyroidism may adversely affect lung development, including impairment of ventilatory responses (71). Previous studies have also shown compromised maternal thyroid function to be associated with lower birth weight (70, 72), which in turn have been suggested to increase the risk of infant asthma (73). Another potential explanation for how iodine could be associated with asthma risk is that seafood, in addition to being a source of iodine as shown herein, also contains long-chain polyunsaturated fatty acids which are known for their immunoregulatory effects (74). Hence, the effects seen for iodine might not be of causal origin. To note, a great majority of the children with asthma diagnosis in the present study had multiple respiratory infections during the first year of life, making the association between iodine intake and asthma at 12 months of age highly uncertain. Hence, it is important to follow up these findings with asthma diagnoses at an older age.

A major strength of this study is the extensive assessment of maternal iodine and selenium from food, based on repeated dietary questionnaires and status biomarkers, as well as infant status biomarkers (concentrations in blood and urine collected at 4 months of age) in relation to breast milk, maternal status, and infant formula. One limitation is that the previous validation studies of the dietary questionnaire validated the intake of selenium, but not that of iodine (33, 34). In addition, the absolute intake of iodine and selenium *via* sources other than diet (e.g., supplements and iodized salt) are not known. However, we evaluated the total intakes based on the biomarker concentrations. Also, single spot-urine samples were used, which might not reflect habitual iodine intake due to the short body half-life, resulting in large day-to-day and within-day variations (25). However, the women were asked to fast several hours before sampling, and we adjusted UIC for variation in urine dilution. Also, the observed correlation of maternal UIC with breast milk iodine, despite the seven months in between the sample collections, indicates reasonably reliable estimates. A 24 h urine collection would cause a higher burden for the pregnant and lactating women, and such samples are often incomplete (75). Another weakness of the study is the lack of data on iodine and selenium status at birth, which would have expanded the information on placental vs. breast milk transfer of selenium. Also, infant plasma selenium would have been useful.

## Conclusions

Our results indicate that the transport of iodine and selenium to the fetus and infant are prioritized, and the maternal iodine and selenium status seemed inadequate. The generally low intake of iodine and selenium in both mothers and their infants in this Swedish mother-child cohort, especially when the mothers did not use supplements or iodized table salt, do raise concern. A follow-up of the children's nutritional status and related health consequences, including thyroid function and allergy, is warranted. Obviously, there is a need of interventions targeting pregnant and lactating women, primarily focusing on promoting an increased intake of fish, dairy products, and iodized salt. However, even the women using iodized table salt did not reach the recommended group level for sufficient intake, why iodine supplementation needs further consideration, as also proposed by the WHO Technical Consultation for regions where iodine nutrition is inadequate (45). Importantly, with an increased consumption of seafood it is important to promote sustainable sources and consider the type and source of origin wisely to avoid exposure to environmental pollutants (76).

## Data Availability Statement

The datasets presented in this article are not readily available because they relate to information that could compromise research participant privacy or consent. Explicit consent to deposit raw data was not obtained from the participants. Therefore, the data can only be made public if a new consent is filled in by the participants together with a new ethical permit being obtained. The R-codes for conducting the Spearman correlation, drawing the heatmaps, creating the correlation plots, creating the histograms, and conducting the logistic regression, can be obtained from: https://gitlab.com/miastravik/iodine-and-selenium. Requests to access the datasets should be directed to Maria Kippler, maria.kippler@ki.se.

## Ethics Statement

The study involved human participants and was reviewed and approved by Regional Ethical Review Board in Umeå, Sweden (2013/18–31M, 2015–71–32) and conducted in accordance with the Helsinki Declaration. Written informed consent to participate in this study was provided by the participants' legal guardian/next of kin.

## Author Contributions

MV, MK, MB, A-SS, AEW, and AS: conceptualization. MS, KG, and MK: data curation. MS: formal analysis, visualization, and writing—original draft. MS, MK, KG, and AS: investigation. MB, A-SS, MK, and MV: methodology and supervision. MB, AS, A-SS, MK, and MV: project administration. MS, MV, MK, MB, KG, HS, A-SS, AS, and AEW: writing—review and editing. All authors have read and agreed to the published version of the manuscript.

## Funding

The authors declare that this study received funding from Swedish Research Council (FORMAS) (2019-01007, MV); Swedish Research Council (FORMAS) (2018-02275, MK); Swedish Research Council (Vetenskapsrådet) (521-2013-3154 and 2019-01317, A-SS); Swedish Research Council for Health, Working Life and Welfare (FORTE) (2014-0923 and 2018-00485, AEW); Västra Götaland Region (RUN) (612–0618–15, A-SS); Research and Innovation Unit at Region Norrbotten (AS); Dr. P.Håkansson's Foundation, Eslöv, Sweden (MB) and Jane och Dan Olssons stiftelse (2020–23, A-SS). The funders were not involved in the study design, collection, analysis, interpretation of data, the writing of this article, or the decision to submit it for publication.

## Conflict of Interest

The authors declare that the research was conducted in the absence of any commercial or financial relationships that could be construed as a potential conflict of interest.

## Publisher's Note

All claims expressed in this article are solely those of the authors and do not necessarily represent those of their affiliated organizations, or those of the publisher, the editors and the reviewers. Any product that may be evaluated in this article, or claim that may be made by its manufacturer, is not guaranteed or endorsed by the publisher.
