# Supplementary material for: Infant Iodine and Selenium Status in Relation to Maternal Status and Diet During Pregnancy and Lactation
Source: Front Nutr. 2021 Dec 17;8:733602. doi: 10.3389/fnut.2021.733602 (PMC8721874; doi:10.3389/fnut.2021.733602)
Supplement: Supplementary file 1 [file Data_Sheet_1.pdf]

Supplementary Material

1. Supplementary Tables

Supplementary Table 1. Reference material for analysis of maternal blood and urine from gestational week 29.

Quality control	n	Reference material	Reference value (reference interval) (µg/L)	Obtained mean (SD) (µg/L)
UIC	51	Seronorm Urine 1011644 L-1	84 (72-96)	76 (7.8)
UIC	52	Seronorm Urine 1011645 L-2	304 (260-348)	264 (25)
Ery-Se	21	Seronorm whole blood-1406263 L-1	60 (48-72)	58 (5.8)[a]
Ery-Se	28	Seronorm whole blood-1406264 L-2	161 (128-193)	152 (17.5)[a]
P-Se	19	Medisafe serum 28341	50 (41-59)	54 (4.9)[b]
P-Se	10	NIST serum 1598a	134.4 (5.8)	144 (18.0)[b]

[a]The presented values have been adjusted to the assumed whole blood density of 1.055 kg/L (1) to convert from the measured unit of µg/kg to the unit of µg/L.
[b]The presented values have been adjusted to the assumed serum density of 1.026 kg/L (1) to convert from the measured unit of µg/kg to the unit of µg/L.

Supplementary Table 2. Reference material for analysis of breast milk and infant urine and erythrocytes at four monthsof age.

Quality control	n	Reference material	Reference value (reference interval) (µg/L)	Obtained mean (SD) (µg/L)
Ery-Se	12	Seronorm whole blood 1702821 L-1	69 (51-80)	61 (7.5)[a]
Ery-Se	12	Seronorm whole blood 1702825 L-2	144 (113-175)	126 (15)[a]
UIC	15	Seronorm Urine 1011644-L1	84 (72-96)	84 (4.8)
BM-Se	27	Seronorm whole blood -1702821 L-1	69 (51-80)	634 (5.3)[a]
BM-Se	27	Seronorm whole blood -1702821 L-2	144 (113-175)	125 (14.9)[a]
BM-Se[b]	27	NIST Infant Formula 1849	889[c]	1018 (120)
BM-I	27	Seronorm whole blood -1702821 L-1	24 (19-29)	27 (4.8)[a]
BM-I	27	Seronorm whole blood -1702821 L-2	63 (50-75)	61 (5.8)[a]
BM-I[b]	27	NIST Infant Formula 1849	1370[c]	1389 (130)

[a] The presented values have been adjusted to the assumed whole blood density of 1.055 kg/L (1) to convert from the measured unit of µg/kg to the unit of µg/L used for the reference values.
[b] Concentrations in µg/kg.
[c] Certified concentration.

References

1. Lentner C. Geigy Scientific tables. Vol. 3, Physical chemistry, composition of blood, hematology, somatomeric data. Basle: Ciba-Geigy; 1984.

Supplementary Table 3. Biomarker iodine and selenium concentrations in gestational week 29 (GW29) and at fourmonths postpartum (4M) grouped by extent of breastfeeding between three and four months.

Biomarker	Never (n=71)		Partially (n=117)		Exclusively (n=342)		
	n	Median (5th-95th)	*n*	Median (5th-95th)	*n*	Median (5th-95th)	p[1]
**Iodine**							
Breast milk, µg/kg (4M)	*0*	-	*73*	64.5 (23.7-212)	*250*	77.4 (35.6-194)	0.096
Infant urine, µg/L (4M)	*47*	125 (89.2-228)[a]	*71*	115 (43.3-233)	*224*	105 (47.0-248)[a]	0.006
**Selenium**							
Breast milk, µg/kg (4M)	*0*	-	*73*	9.00 (5.73-15.3)	*250*	9.00 (5.67-12.9)	0.718
Infant erythrocytes, µg/kg (4M)	*33*	97.0 (78.1-140)	*45*	91.1 (74.7-128)	*141*	97.4 (72.2-135)	0.614

[1] Differences between the three groups were tested with Kruskal-Wallis test, with the exception of concentrations in breast milk which were tested with Mann-Whitney U test between the groups "partially" and "exclusively". In addition, Mann-Whitney U test was used as a post-hoc test and significant differences are displayed with matching letters in superscript.

Information regarding extent of breastfeeding was collected primarily from questionnaires sent out every month during the child's first year of life, asking: "Did you breastfeed during the past month and if so, to what extent, not at all, partly, exclusively?". The replies from the fourth questionnaire were used when available (n=414) since that reflects the breastfeeding habits between month three and four (when selenium and iodine were measured). For the 190 women who did not reply at that specific month, replies in month five were used as a proxy for month four if any extent of breastfeeding was reported that month (n=53). In cases of reply in month five that they did not breastfeed to any extent, exclusive breastfeeding in month three was used as a proxy for partial breastfeeding in month four (n=2), and no breastfeeding in month three was used as a proxy for no breastfeeding in month four (n=4). For the remaining 131 women, any report of exclusive breastfeeding in month six and thereafter was used as a proxy for exclusive breastfeeding also in month four (n=7). At this stage, 124 women were still not categorized, and information was therefore collected from a dietary questionnaire sent out when the child reached twelve months of age, including the question: "For how long was your child exclusively breastfed (without any other food or drink)?": 0-2, 3-4, 5-6, >6 months. If duration was reported to be ≥3-4 months they were considered to breastfeed exclusively in month four (n=40). For the remaining 84 women, another question from the same questionnaire was used: "Is your child currently being breastfed?": yes, no but previously, never. If they replied "never" at twelve months of age, this was used as a proxy for no breastfeeding in month four (n=3). As a last step, information from another question in the one-year questionnaire was used: "For how long was your child breastfed (partly or exclusively)?" with the alternatives: <6, 6-8, 9-12, 13-18, >18. If duration was reported to be ≥6-8 months (n=4), they were assumed to be partially breastfeeding in month four. Among the ones classified as "never" breastfed in month four, 11 women still had available breast milk samples. These were therefore assumed to be breastfeeding to some extent and moved to the group "partially". In addition, 12 women in the non-classified category (n=77) had available breastmilk samples from month four. These were investigated further, one-by-one. In 3 of these cases, duration of exclusive breastfeeding was reported as 0-2 months from the questionnaire sent out at twelve months. Since breastmilk samples were obtained at four months, these cases were coded as partially breastfeeding between month three and four. In 5 cases, there was no available information, neither from the twelve questionnaires sent out monthly during the first year of life, nor from the questionnaire sent out at twelve months of age. In the remaining 4 cases, a reasonable distinction between partially or exclusively breastfeeding between month three and four could not be made based on available data. Hence, these 9 women were excluded from analyses regarding breastfeeding.

Supplementary Table 4. Biomarker iodine and selenium concentrations in gestational week 29 (GW29), gestational week 34 (GW34) and at four months postpartum(4M) grouped by allergy diagnosis of the child at 12 months of age.

Biomarker	*n*	Non-allergic	*n*	Food allergy	*n*	Atopic eczema	*n*	Asthma
		**Median (25th-75th)**		**Median (25th-75th)**		**Median (25th-75th)**		**Median (25th-75th)**
**Iodine**								
Dietary intake, µg/d (GW34)	*370*	101 (70.6-140)	*38*	85.6 (53.1-131)	*32*	89.6 (57.0-146)	32	85.2 (57.3-144)
Maternal urine, µg/L (GW29)[1]	*380*	112 (81.8-155)	*38*	118 (83.9-207)	*33*	144 (96.6-203)	34	111 (74.7-134)
Dietary intake, µg/d (4M)	*373*	112 (78.2-150)	*37*	103 (53.7-143)	*31*	105 (65.6-160)	32	80.8 (53.2-134)*
Breast milk, µg/kg (4M)	*256*	77.7 (50.5-120)	*23*	72.5 (62.0-116)	*20*	65.4 (48.6-94.1)	16	70.0 (50.0-81.0)
Infant urine, µg/L (4M)[2]	*266*	114 (80.6-150)	*25*	117 (92.0-132)	*21*	110 (91.0-161)	23	114 (81.5-149)
Breastfed[3]	*182*	112 (73.3-150)	*13*	117 (98.8-188)	*11*	107 (91.9-172)	8	99.5 (63.1-134)
Not breastfed[3]	*84*	117 (93.7-145)	*12*	113 (86.0-127)	*10*	116 (89.7-160)	15	120 (110-155)
**Selenium**								
Dietary intake, µg/d (GW34)	*370*	40.2 (30.1-51.9)	*38*	38.5 (24.1-49.3)	*32*	37.5 (25.4-49.8)	32	39.2 (26.6-52.9)
Maternal plasma, µg/kg (GW29)	*380*	64.9 (57.6-74.6)	*39*	69.0 (61.0-75.6)	*33*	65.7 (59.1-74.1)	34	68.3 (59.8-75.0)
Maternal erythrocytes, µg/kg (GW29)	*379*	106 (94.9-119)	*40*	103 (92.9-119)	*34*	104 (91.8-121)	34	109 (91.3-119)
Dietary intake, µg/d (GW34)	*373*	39.0 (29.9-52.4)	*37*	33.1 (23.3-43.9)	*31*	39.1 (31.4-52.6)	32	34.6 (25.8-49.2)
Breast milk, µg/kg (4M)	*256*	8.95 (7.48-10.2)	*23*	9.57 (6.93-10.7)	*20*	9.51 (6.88-10.8)	16	9.15 (7.88-11.0)
Infant erythrocytes, µg/kg (4M)	*166*	97.4 (87.8-113)	*18*	93.4 (85.0-102)	*18*	94.7 (79.8-104)	8	98.2 (91.2-131)
Breastfed[3]	*107*	97.5 (86.3-114)	*11*	90.9 (83.0-101)	*9*	95.2 (75.7-116)	7	96.6 (90.9-132)
Not breastfed[3]	*59*	97.4 (89.4-109)	*7*	95.8 (86.3-103)	*9*	94.3 (85.5-99.1)	1	99.8 (NA)

Allergy was diagnosed by the study pediatrician at twelve months of age. Differences between each allergy group and the non-allergic group were tested with Mann-Whitney U test and significant differences are denoted with asterisks as follows: p<0.001=***, p<0.01=** and p<0.05=*.

[1] Adjusted to the mothers mean urinary specific gravity (SG_{mean}=1.017).

[2] Adjusted to the infants mean urinary specific gravity (SG_{mean}=1.004).

[3] Individuals with available breast milk samples at four months were defined as being breastfed

2. Supplementary Figures

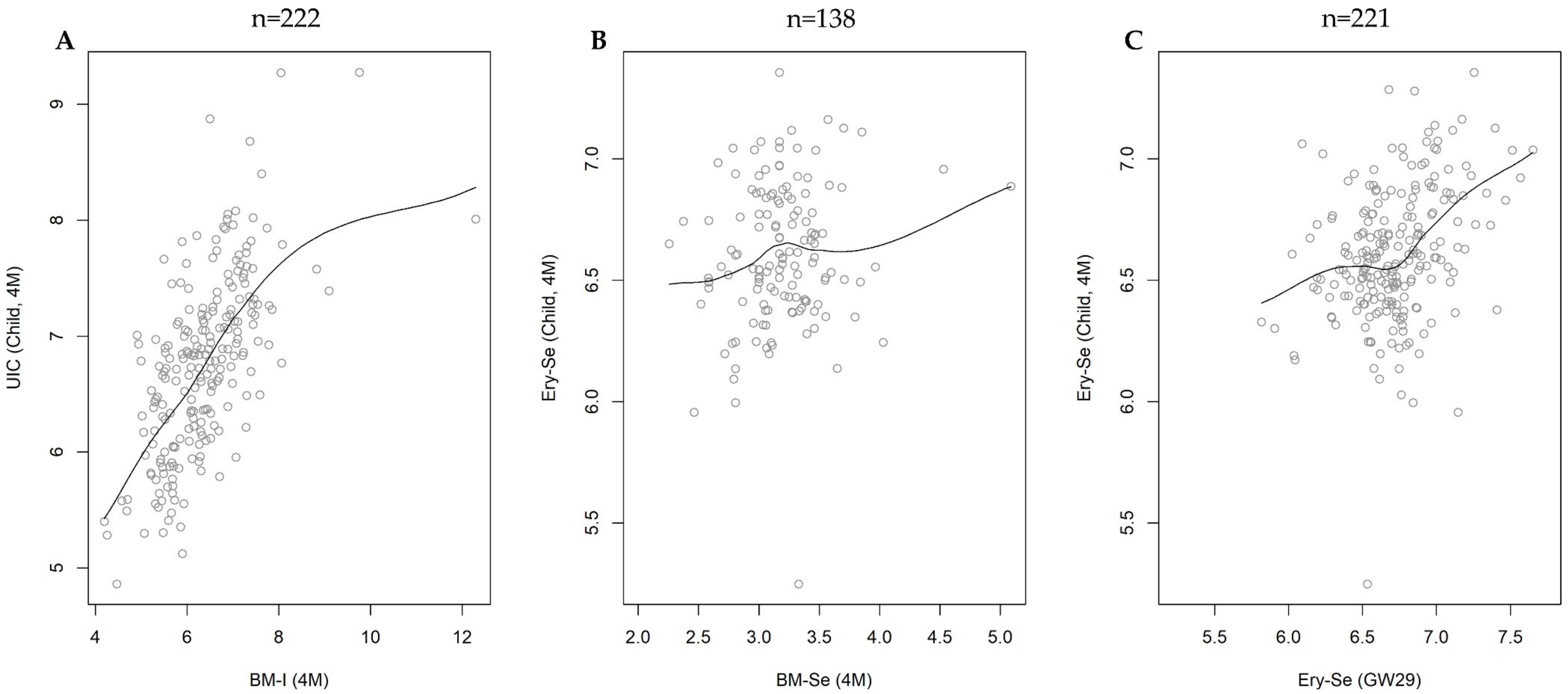

Supplementary Figure 1. Scatter plots with LOWESS smoothing curve of log2-transformed concentrations of **A)** iodine in breast milk (µg/kg) with breastfed infant's urine (µg/L), **B)** selenium in breast milk (µg/kg) in relation to breastfed infant's erythrocytes (µg/kg), and **C)** selenium in infant's erythrocytes in relation to selenium in maternal erythrocytes (µg/kg).

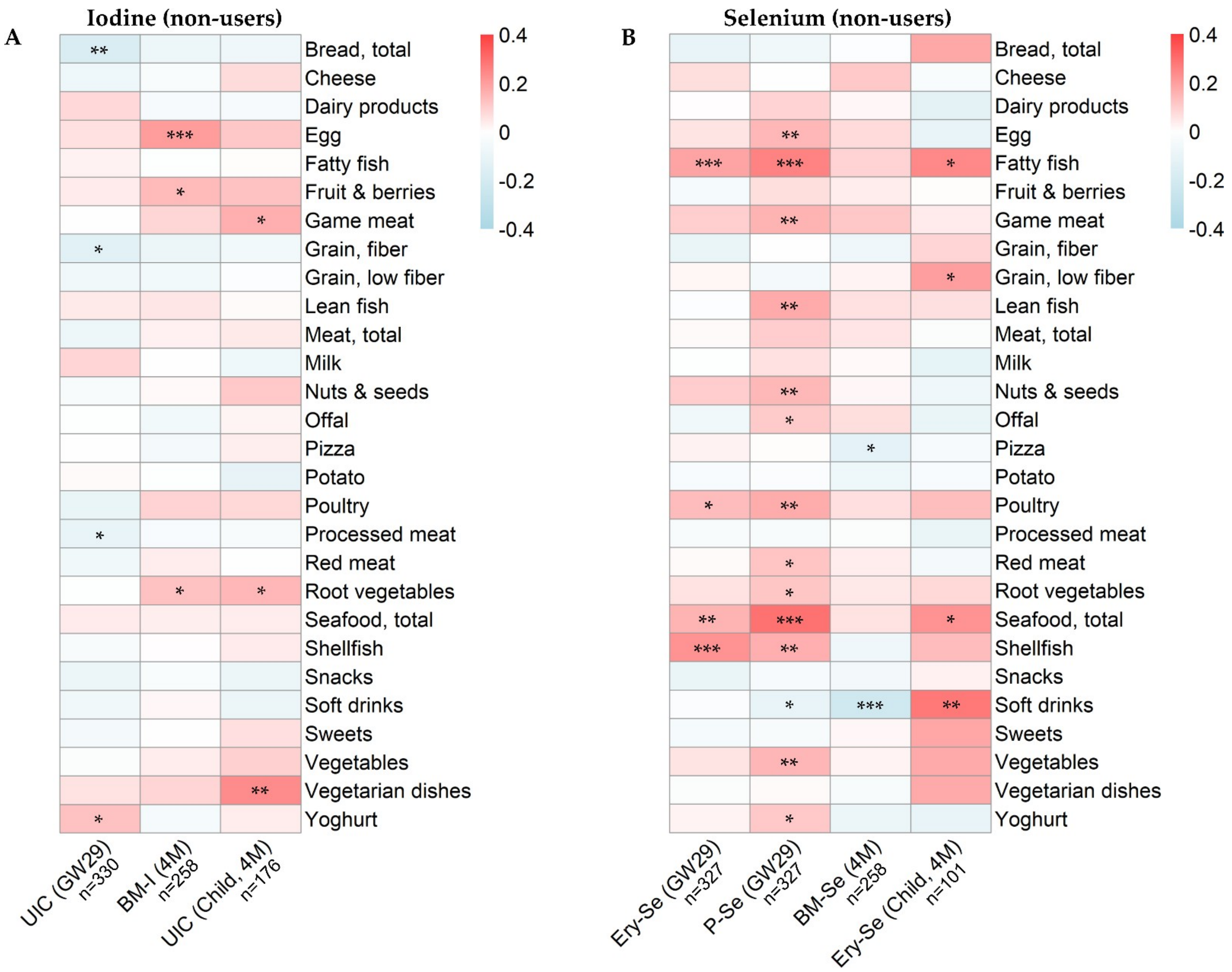

Supplementary Figure 2. Heatmaps displaying Spearman correlations between **A)** iodine concentrations in urine (UIC) and breast milk (BM-I), and maternal food intake among women *not* taking multivitamins with minerals regularly, and **B)** selenium concentrations in erythrocytes (Ery-Se), plasma (P-Se) and breastmilk (BM-Se), and maternal food intake among women not taking multivitamins with minerals regularly. Food intake was assessed to reflect maternal intake in gestational week 30-34 and at 3-4 months postpartum. Associations between infants' iodine and selenium concentrations and maternal food intake was investigated solely for breastfed infants (i.e., with available breast milk samples). Significant correlations are denoted with asterisks as follows: p<0.001=***, p<0.01=** and p<0.05=*.
